# When there is a pandemic there is no time to waste: should we have hydroxychloroquine in our armoury against COVID-19 infected patients?

**DOI:** 10.31138/mjr.31.1.94

**Published:** 2020-03-31

**Authors:** Dimitrios P. Bogdanos, Zoi Daniil, Epaminondas Zakynthinos, Konstantinos Gourgoulianis, Lazaros I. Sakkas

**Affiliations:** 1 Department of Rheumatology and Clinical Immunology,; 2 Department of Respiratory Medicine, and; 3 Intensive Care Unit, Faculty of Medicine, School of Health Sciences, University of Thessaly, Larissa, Greece

**Keywords:** pandemic, chloroquine, hydroxychloroquine, COVID19

## Abstract

The current use of chloroquine and/or hydroxychloroquine, a drug currently used to treat autoimmune rheumatic diseases, in treating severe acute respiratory syndrome caused by coronavirus 2 (SARSCoV-2) or COVID-19-infected patients with pneumonia is a matter of intense consideration. We wish to enter the ongoing debate as to whether this well-known drug must be given to Greek COVID-19-infected patients, especially those with pneumonia. Our arguments are based on the existing data and the capacity of the Greek health system to afford potent anti-viral treatments, which are under immense investigation. We propose several suggestions related to treatment of COVID-19 pneumonia with chloroquine/hydroxychloroquine that we think must be taken into consideration to fit the evolving situation of the pandemic in Greece.

There is no certainty that chloroquine and/or hydroxychloroquine is efficacious for the severe acute respiratory syndrome caused by coronavirus 2 (SARS-CoV-2) or COVID-19-infected patients with pneumonia, especially immunocompromised patients. We wish to enter to the ongoing debate as to whether this well-known drug must be given to Greek COVID-19-infected patients, especially those with pneumonia.

The available data thus far are limited, originate mainly from China, and are inadequate to reach to a safe conclusion. It is generally accepted that no medication should be given to a patient unless it causes much more good than harm. But at times of crisis, as most leaders of the world state with regard to the COVID-19 pandemic, such rules may not apply (https://www.bbc.com/news/world-us-canada-51955450). Based on the Italian and Spanish experience, we anticipate that we will have a shortage of ventilators and intensive care unit beds if we have many severe COVID-19-infected cases in Greece ( https://www.reuters.com/article/us-health-coronavirus-draegerwerk-ventil/germany-italy-rush-to-buy-life-saving-ventilators-as-manufacturers-warn-of-shortages-idUSKBN210362 ).

We may even be in shortage of liquid oxygen due to huge demands of hospitalized patients, as we have seen in other countries (https://www.express.co.uk/news/world/1256411/Coronavirus-UK-news-Italy-update-latest-hospitals-NHS).

Hence, there is no time to waste. We must probably be proactive and ready to take action. Most of the available therapeutic tools are not cost-effective, especially for countries like Greece, which has experienced a fiscal crisis for years. Amongst those, only one is economical and affordable for the Greek health system: chloroquine and hydroxychloroquine, a drug belonging to 4-amino-quinoline class. Its efficacy and safety profile may not be desirable, but it may be placed on the top of the respective list of available anti-viral agents.

Do we really need to wait for international guidelines to be issued regarding its applicability to COVID-19 pneumonia? This may take weeks or even months, as the available data are still limited, and large clinical trials are still at the beginning. We are running out of time. We may need to be as proactive, as our Chinese colleagues have been so far. In China alone, at least 23 clinical trials investigate the efficacy and safety of chloroquine or hydroxychloroquine for the treatment of COVID-19-associated pneumonia.^1^ These studies are conducted in several hospitals treating thousands of COVID-19 infected patients in large cities such as Wuhan, Jingzhou, Guangzhou, Beijing, Shanghai, Chongqing, and Ningbo.^1^. Limited data from Europe, such as those from France, are encouraging.^2^ A clinical trial of this kind, investigating amongst other the immunogenicity of the drug, is also going to start soon at the University Hospital of Larissa, Central Greece. Other hospitals in Greece are anticipated to follow.

Based on the Chinese data,^3^ chloroquine phosphate is superior to control treatment in promoting a virus-negative conversion and most importantly in constraining the exacerbation of pneumonia, improving lung imaging findings, and limiting disease course duration. The data are not definite and thousands of Chinese patients are still to be included for the final verdict. In China, the experts have been gathered, have analysed the existing data and have reached a decision. The National Health Commission of the People’s Republic of China has issued guidelines, and chloroquine is now recommended for the treatment of pneumonia caused by COVID-19 as per 6^th^ and 7^th^ Edition of the Guidelines for the Prevention, Diagnosis, and Treatment of Pneumonia Caused by COVID-19. The expert panel of the National Public Health Authority has very recently (26 March 2020) revised the therapeutic protocol/algorithm for the treatment of symptomatic COVID-19 patients, which includes hydroxychloroquine/chloroquine.

A harmonized pan-European protocol issued by the European health authorities is urgently needed. China, Italy (Lombardy protocol), the Netherlands, Belgium, Switzerland, France and other countries have issued specific therapeutic protocols based on the stratification of patients. However, in existing recommendations, there is no consensus on dosage, duration and patient selection for therapeutic intervention. Each country follows the recommendations issued by their respective Expert panel and Health Authority Task Force, and significant discrepancies exist amongst therapeutic protocols. In some countries, these recommendations are updated in a regular basis, taking into account the evolving data.

Of relevance, the World Health Organisation (WHO) is launching SOLIDARITY, a mega-trial recruiting thousands of patients all over the globe to assess the efficacy of all existing anti-viral agents used to treat COVID-19 patients as reported in Science: “enrolling subjects in SOLIDARITY will be easy. When a person with a confirmed case of COVID-19 is deemed eligible, the physician can enter the patient’s data into a WHO website, including any underlying condition that could alter the course of the disease. After the physician states which drugs are available at his or her hospital, the website will randomize the patient to one of the drugs available or to the local standard care for COVID-19” (https://www.sciencemag.org/news/2020/03/who-launches-global-megatrial-four-most-promising-coronavirus-treatments ).

In Europe, several studies have started recruiting patients to assess the efficacy and safety of the drug. Based on clinicaltrial.gov repository, the Norwegian Coronavirus Disease 2019 Study (NOCOVID-19) was launched on March 23 (NCT04316377). This is a two-arm, open label, pragmatic randomized controlled trial which is anticipated to enrol approximately 200 patients. In this trial, hydroxychloroquine Sulphate (Plaquenil) 400 mg will be given twice daily for seven days. The primary outcome of this trial is the Rate of decline in SARS-CoV-2 viral load (Baseline [at randomization] and at 96 hours). Secondary outcomes include Change in National Early Warning Score, admission to intensive care unit, in-hospital mortality, duration of hospital admission, mortality at 30 and 90 days and clinical status.

Even if we start treating COVID-19 pneumonia patients with this drug several questions remain unanswered. Who is going to be treated? When must we initiate treatment? Do we need to stratify patients according to severity or not? Can it be given as a prophylaxis, as in the case of malaria? What is the appropriate dose and what is the ideal duration of treatment?

We have noted that not all Chinese clinical trials conducted so far are using the same dosage and treatment duration. Nevertheless, according to the recently issued 7^th^ edition of the formal Chinese guidelines, chloroquine phosphate is suitable for the treatment of COVID-19 pneumonia in adults aged 18 to 65 years old. For those weighing more than 50 kg, the course of treatment is 500 mg twice per day for 7 days, while for those weighting 50 kg or less, the course of treatment is 500 mg twice a day on day 1 and day 2, followed by 500 mg given once per day on days 3 to 7 (http://www.gov.cn/zhengce/zhengceku/2020-03/04/5486705/files/ae61004f930d47598711a0d4cbf874a9.pdf)

The most important issue is the safety of the drug at the proposed doses. Statements in the Greek media such as “the drug is extremely toxic and must not be given because it can kill people” (https://www.ethnos.gr/ella-da/94961_exalli-i-epistimoniki-koinotita-afiste-ti-hlorokini-tha-sas-skotosei) are not helpful. The Chinese Guidelines are very explicit concerning adverse effects, precautions, and contraindications. Most of those are well-known and are reported in the summary product characteristics (SPC) (HCQ phosphate, Plaquenil, Sanofi-Aventis) (http://products.sanofi.ca/en/plaquenil.pdf) and relate to: pregnancy; allergy to 4-aminoquinolines; cardiac arrhythmias (such as heart block); chronic heart disease; chronic liver or kidney diseases that reach terminal stages; a history of retinal disease, hearing loss or deafness; history of mental illness; skin diseases (including rash, dermatitis, and psoriasis); glucose-6-phosphate dehydrogenase deficiency; concurrent usage of the following for pre-existing conditions: digoxin and derivatives, phenylbutazone, heparin, penicillamine, amiodarone, bepridil, domperidone, droperidol, haloperidol, azithromycin, astemizole, erythromycin, clarithromycin, posaconazole, methadone, procainamide, hydrochlorothiazide, sparfloxacin, levofloxacin, moxifloxacin, cisapride, indapamide, chlorpromazine, streptomycin, penicillamine, ammonium chloride, ondansetron, apomorphine, octreotide, MAO inhibitors, and triamcinolone ( http://www.gov.cn/zhengce/zhengceku/2020-03/04/5486705/files/ae61004f930d47598711a0d4cbf874a9.pdf )

Precautions when using chloroquine phosphate to treat patients with COVID-19 pneumonia are also described in the Chinese guidelines: electrocardiogram must be normal prior to medication, and the simultaneous use of quinolones, macrolide antibiotics and other drugs that may cause QT interval prolongation is prohibited. The patient should have stable potassium and sodium levels, normal blood sugar, and normal liver and kidney function. Special attention must be paid to potential drug interactions. Using 3 or more antiviral drugs (including chloroquine phosphate) is not recommended. There should be close monitoring for adverse drug reactions and discontinuation of the drug if intolerable adverse reaction develops (http://www.gov.cn/zhengce/zhengceku/2020-03/04/5486705/files/ae61004f-930d47598711a0d4cbf874a9.pdf)

Hydroxychloroquine has been used for decades to prevent and treat malaria (it was first used during World War II) and is also effective as an anti-inflammatory and immunomodulatory agent for the management of various autoimmune rheumatic diseases, such as rheumatoid arthritis, Sjögren’s syndrome, systemic lupus erythematosus, and even sarcoidosis. Thousands of Greek patients with rheumatic diseases are currently treated with hydroxychloroquine (200 mg or 400 mg per day) for months or even years. Millions of people with the respective rheumatic diseases are receiving this drug all over the world. Severe adverse reactions are limited, and only a minor proportion of the patients that have an indication to take the drug had to discontinue it because of adverse reactions (for more information visit https://www.ncbi.nlm.nih.gov/books/NBK537086/). We have a long experience with the drug, its safety profile and its efficacy for the treatment of patients with rheumatic diseases and co-existing comorbidities, and we are currently treating hundreds of patients in Central Greece with this medication. Having said that, caution must be exercised for over-the-counter use of medications. The American media reported that a man in the Phoenix area has died and his wife was in critical condition after the couple took chloroquine phosphate, an additive used to clean fish tanks (https://edition.cnn.com/2020/03/23/health/arizona-coronavirus-chloroquine-death/index.html ).

The safety profile of the drug specifically for COVID-19 treated patients is also a topic of intense research. The COPCOV French observational study will recruit 1000 participants and will address adverse events related to treatments used against SARS-CoV-2, including chloroquine. In UK, a Randomised, Placebo-controlled Prophylaxis Study (NCT04303507) will assess the prophylactic effect of chloroquine (Chloroquine Prevention of Coronavirus Disease [COVID-19] in the Healthcare Setting). This study is a double-blind, randomized, placebo-controlled trial that will be conducted in health care settings. It will include healthcare workers or other individuals at significant risk, who can be followed up for 5 months. 10,000 participants will be recruited, and the investigators predict an average of 200 participants per site in 50 sites. A loading dose of 10mg/kg of body weight will be given, followed by 150 mg daily (250mg chloroquine phosphate salt), which will be taken for 3 months or until they are diagnosed with COVID-19. Subsequent episodes of symptomatic respiratory illness, including symptomatic COVID-19, clinical outcomes, and asymptomatic COVID-19 infection will be recorded during the follow-up period.

Clinical trials of this kind are initiated based on the urgent need for prompt anti-viral treatment and preliminary *in vitro* and *in vivo* data. Some experts think hydroxychloroquine is a promising start for the treatment of COVID-19-related pneumonia.

The drug’s anti-coronavirus activity has been demonstrated.^4–9^ Hydroxychloroquine appears to be more potent *in vitro* inhibitor than chloroquine in inhibiting SARS-CoV-2 infected Vero cells^10^ and inhibits HCoV-OC43 infected HRT-18 cells.^7^ These data have also been corroborated by findings in infected mice,^7^ but we still miss data on SARS-CoV-2. It also looks like chloroquine also exerts its anti-viral action by increasing endosomal pH required for virus/cell fusion,^5,11^ It also interferes with the glycosylation of cellular receptors of SARS-CoV and this may at least in part explain its potent efficacy in treating patients with COVID-19 pneumonia.^5^ Chloroquine may also have an anti-thrombotic effect in COVID-19 patients appearing with vasculitides or thrombotic episodes. It may also play an inhibitory role in the cytokine storm phenomenon noted in COVID-19 ARDS through the abrogation of pro-inflammatory cytokine release. It is not clear though what the effect of the drug is on antigen-specific humoral and cellular immune responses against COVID-19 immunodominant antigens.

**We suggest the following initiatives:**
1. The Greek Health Authorities must secure large amounts of hydroxychloroquine/chloroquine, sufficient enough to cover the country’s needs before the extreme amplification phase of the pandemic. Very recently, a Greek pharmaceutical company imported a large amount of chloroquine and will provide, for free, 24 million doses to the Greek Health authorities for general hospital needs ( http://www.ekathimerini.com/251072/article/ekathimerini/news/uni-pharmato-produce-offer-free-chloroquine-to-state-for-covid-19-treatment). This is very encouraging.
2. Its use must be restricted to hospitalised patients.
3. Synoptic tables must be drafted from the national Health Authorities summarizing the selected investigational drugs to be consider for clinical use at this moment with information on in *vitro/in vivo* efficacy, the current therapeutic recommendations for each category of COVID-19 patients, with indications and precautions, and the treatment protocols. An excellent example of such tables is probably that prepared by the Belgium Task Force (*Interim clinical guidance for patients suspected of/confirmed with COVID-19 in Belgium [19 March 2020. Version 4]*).
4. A public Campaign must be initiated ensuring the Greek public that the drug is available in sufficient amounts to calm public anxiety.
5. Greek Medical Professionals must thoroughly and regularly be informed about its applicability in treating COVID-19 pneumonia through the respective medical societies and professional associations.
6. Meticulous attempts need to be made for as many as possible Greek COVID-19 patients to be included in the SOLIDARITY trial or other pan-European, multi-international initiatives.
7. National clinical trials on the efficacy, safety and antigenicity and anti-viral efficacy of this regimen in Greek patients with COVID-19 must be initiated, as soon as possible.
8. Registries of patients with autoimmune rheumatic diseases treated with plaquenil, infected or not with COVID-19 must be formulated as soon as possible.
9. Fast-track grant schemes providing support for translational and basic research on the topic must be initiated from National Grant Giving Bodies.
10. A national biobanking facility must be initiated, collecting biomaterial for current or future translational research in Greek COVID-19 patients.

In conclusion, the question of whether (hydroxy)chloroquine is efficacious for the treatment of COVID-19 associated pneumonia, will be answered in the not too distant future. In fact, by the time this article is published, we will probably have some answers.
